# Strength in numbers: patient experiences of group exercise within hospice palliative care

**DOI:** 10.1186/s12904-016-0173-9

**Published:** 2016-12-13

**Authors:** Lorna Malcolm, Gill Mein, Alison Jones, Helena Talbot-Rice, Matthew Maddocks, Katherine Bristowe

**Affiliations:** 1 St Christopher’s Hospice, 51-59 Lawrie Park Road, Sydenham, London, SE26 6DZ UK; 2 Faculty of Health, Social Care and Education, St George’s University of London and Kingston University, Cranmer Terrace, London, SW17 0RE UK; 3 King’s College London, Cicely Saunders Institute, Division of Palliative Care, Policy & Rehabilitation, Bessemer Road, London, UK

**Keywords:** Hospice, Palliative care, Palliative rehabilitation, Exercise, Group exercise, Patient experience, Qualitative, Phenomenology

## Abstract

**Background:**

Exercise is increasingly recognized as a core component of palliative rehabilitation. The group exercise model is often adopted as a means of reaching more patients with limited resource. Despite the growth of quantitative research examining this area of practice, few qualitative studies have looked at the patient experience of participating in group exercise in a palliative setting, and most exclude patients with a non-cancer diagnosis.

**Methods:**

The aim of this study was to explore patients’ experiences of participating in group exercise classes in a hospice setting. In this qualitative, phenomenological study, nine patients participating in a group exercise programme at a South London hospice completed semi-structured interviews. Participants were purposively sampled by gender, age, ethnicity and diagnosis; to include diagnoses across cancer, respiratory and neurological conditions. Transcripts were interpreted using thematic analysis.

**Results:**

All patients reported positive experiences of participating in group exercise classes. Improvements reported in physical function had a positive effect on ability to complete activities of daily living and enhanced patient mood. Other reported psychosocial benefits included: promotion of self-management; space and opportunity for reflection; supportive relationships; sharing of information; and a deeper appreciation of patients’ own abilities.

**Conclusion:**

This study highlights the positive experiences and value of group exercise classes to groups of people with diverse cancer and non-cancer conditions. The physical, emotional and psychosocial benefits suggest hospices and other palliative services should explore similar programmes as part of their rehabilitation services. The recognition that exercise groups can be mixed and need not be bespoke to one condition has positive cost and staff resource ramifications.

## Background

In recent years the scope of practice for palliative care has expanded. Whilst previously palliative care focused primarily on ensuring comfort for patients during the dying process [1], more recently the value of palliative care has been recognized for people throughout the course of incurable or long term conditions, which may not be immediately terminal [2]. This shift has encouraged a focus on living well with progressive disease, in order to optimize physical capabilities and function irrespective of prognosis. Indeed, rehabilitation is now considered integral to palliative care [3, 4] with exercise a key component therapy [5, 6] from which those with advanced progressive illnesses may benefit [2, 7]. Exercise can help reduce risks of, or manage symptoms of, advanced disease and its treatment; helping improve psychosocial well-being and health-related quality of life [8, 9]. The suggestion that people with cancer should participate in rehabilitation to ‘be productive’ and ‘function at a minimum level of dependency’ [10], is also appropriate for other long term conditions. Exercise is also considered a useful supportive therapy alongside treatments for the underlying disease, offering patients an element of control, an enhanced sense of hope and positivity, and a return to some semblance of normality [11, 12].

The Minimum Data Set for Specialist Palliative Care report [13] showed a large increase in people accessing specialist palliative care services, especially those with non-cancer diagnoses. To adapt and meet the needs of the growing palliative care population, many hospices are adopting group models for delivery of outpatient services [14], including exercise classes [15, 16]. Much of the research relating to palliative rehabilitation is restricted to people with cancer, though evidence from other specialties supports the use of group exercise. For example, pulmonary rehabilitation classes improve symptoms, physical function and quality of life, including in people with advanced illness [17]. A systematic review considering use of group exercise in the treatment of Motor Neurone Disease (MND) was inconclusive [18], though individual studies found physical and psychological benefit, especially when exercise was commenced early in the course of the disease [19]. Qualitative research examining the experience of palliative patients with mixed diagnoses, participating in group exercise is limited [2, 20].

Previous studies have confirmed benefit from participation in exercise for people with cancer, who report a positive experience, but the evidence is limited for groups with multiple diagnoses and/or non-cancer conditions. Understanding the experience of these mixed groups is important as they are more reflective of the different conditions that come under current hospice care. Therefore, the aim of this study was to explore the experience of patients attending group exercise classes at a hospice; particularly in terms of impact upon personal outcomes, management of their illness, as well as perceived function and well-being.

## Methods

### Setting

St Christopher’s Hospice provides specialist palliative care to over 2000 adults annually, from an ethnically diverse population of 1.5 million people across five London boroughs. Group exercise classes have been used as a treatment intervention since the rehabilitation gym was built in 2007. Willing patients with advanced progressive illness, assessed as safe to exercise by the physiotherapy team, come to the gym for weekly, hour-long group sessions of either seated Pilates based exercise or a circuit training class. A recent service evaluation on the circuit training classes found the programme impacts favorably on objective functional outcomes [15].

### Recruitment

Participants were approached between January-June 2014 and purposively sampled, by gender, age, ethnicity and diagnosis to reflect the diverse hospice patient population. Although it was hoped to interview at least 2 patients each with cancer, chronic respiratory disease and MND, only one patient with MND agreed to be interviewed. Potential participants were invited to participate in the study if they had completed at least four group exercise sessions, were fluent in English, and were able to give informed consent.

### Design and analysis

Phenomenology seeks to discover the universal essence of a phenomenon or draw on the experiences of many [21]. Hermeneutic phenomenology allows for the influence of the researcher’s pre-existing experience when creating meaning and developing understanding. The choice of semi-structured interviews, was guided by the aim of gaining insight into patients’ thoughts and feeling about a health service; making their experience visible to others as they perceive it fits into their world; being their description [22] and interpretation of their world [23]. It also stems from acknowledgement that professional perspective, regarding patient experience of a service and the perspective of the patient, may differ.

All interviews were conducted by the lead researcher (LM), a physiotherapist. All participants chose to be interviewed at the hospice, and no participants withdrew from the study after consent was given. The interview topic guide focused on the patients’ experiences of physiotherapy sessions; their experience of participating as part of a group, what they experienced as benefits and negatives of the group sessions; their motivations and expectations for attending; any barriers to attending, and any impact they perceived attending had on their daily lives. Interviews were digitally audio recorded and transcribed verbatim. Interviews were anonymised and pseudonyms allocated for patients and staff when transcribed. Mean interview duration was 34 min (24–55 min).

Interviews were analysed by the researcher using Thematic Analysis, which involves five key stages: familiarisation, coding, theme development, defining themes and reporting. Analysis was supported using an Excel spreadsheet for each interview, to collate codes and consider broader themes. Once coded by the lead researcher, five of the nine interview transcripts were randomly chosen for dual coding by a second researcher to improve reliability, rigour and quality. Transcripts were also returned to participants to confirm the content; one patient made a minor amendment.

## Results

### Participants

The nine interview participants ranged in age from 34 to 93. Five participants had a cancer diagnosis with one also suffering from renal failure, 1 had MND and 3 end stage respiratory conditions. Six participants were female and 3 male; 1 was black African, 5 white British, and 3 black Caribbean, (see Table 1).

**Table 1:** Patient demographics

Interviews	9
Age	
65 and over	5
Under 65	4
Gender	
Female	6
Male	3
Ethnicity	
Black African	1
Black Caribbean	3
White British	5
Disease group	
Cancer	5
Non cancer	4
Time under hospice	
Less than 12 months	5
13–24 months	2
25 months and over	2

### Findings

Three main themes emerged from the data: ‘Perceptions of palliative care and hospice’, ‘supportive relationships’ and ‘taking part in classes’. Eight subthemes were also identified (Table 2).

**Table 2:** Themes and subthemes

Themes	Sub themes	Codes
Perception of hospice	Initial perceptions of hospice	Hospice as a place where people die
	Subsequent perception of hospice	Loss of anxiety about ‘hospice’ once attending regularly
Supportive relationships	Relationships with other patients,	Comparing self with other patients
		Competition with other patients
		Sadness at deterioration/death of others/own mortality
		Receive a ‘boost’
		‘Boring’ if on own
		Being with others in similar position
		Sharing experiences/information
	Relationships with staff	Support/guidance of staff
		Being ‘pushed’/encouraged by staff
		Permission ‘not to have to do’/protected
Taking part in classes	Physical effect	Ability to do things, maintaining current level, not returning to previous disability
		Back to previous self/ doing things used to do before illness
		Wanting to improve strength/fitness
		Independence
		Exercises at home
		Tiredness, aches
		Working to own level/exercises adapted /progressed gradually/ No. in group and time on equipment
	Physical/ psychosocial effect	Therapeutic
	Psychosocial and emotional effect	‘Day out’
		Improved mood/given a boost
		Humour
		Increased confidence/Self esteem/self worth
		Opportunity to reflect
		Coping
	Expectations	Response to suggestion of class participation
		Expectations/not knowing what to expect in classes

### Perception of hospice

Participants shared initial fears and anxiety stemming from their perception of hospice as a place where people go to die:

*“Well the word ‘palliative’ frightened me. Straight away I said to my husband, I don’t think I want that… it’s the end… It meant.... I was coming here for one reason only, the doctors maybe couldn’t do any more for me.... but it meant final.”* (Carol)

These concerns also extended to friends and family, with one participant, Jo, describing avoiding the word ‘hospice’ when talking to her parents, due to the negative connotations they might associate with hospice:

*“…I don’t like saying to my parents ‘I went to the hospice yesterday and did this in physio’ because… I don’t, I don’t want them to hear the word ‘hospice’ and worry… and I think that’s naturally what they’d do.”* (Jo)

However, participants also described a positive change in their perception, attitude or levels of anxiety once they became accustomed to the hospice, particularly once they started attending classes:

*“My mind has totally changed because you can still come here and come out again and live a normal life.”* (George)

Information, explanation and support by the patient’s clinical nurse specialist (CNS), encouragement of family and friends, and actual attendance fostered this adjustment, which took place over varying periods of time.

### Supportive relationships

The majority of patients found relationships with other group members encouraging and supportive; despite observing a deterioration in, and hearing about the death of others:

*“It’s just made me feel happier, you know, joyful… we laugh about things and … it’s just a wonderful feeling being around them.”* (Amy)

Most female patients (but no males) expressed sadness when talking about the deterioration or death of other patients in the group, with some expressing a stoical and/or spiritual attitude to this reminder of their own mortality:

*“Well obviously you feel really sad if its someone you’re fairly close to, and then I suppose at the end of the day, you just think well… as one of the other ladies said who I’m close to, you know, one of these days it’s gonna be us.”* (Fiona)

However, patients described the positive effect on mood and confidence, and the camaraderie engendered by group exercise, as well as it being more enjoyable than exercising alone:

*“I’ve had to sort of … open up a bit [laughs] and be more responsive, … it was very nice coming here because people would greet you, and say hello and ask about you… so I find this time round, when I see somebody I haven’t met, I’m able to go and welcome them.... I’m able to do that in this group situation now, whereas before I would never have… gone up to, you know, someone I don’t know and just start speaking.”* (Emma)

Participants regarded the supportive relationship that developed between group members, as positive, despite concern experienced when a member was absent. A valuable part of the support was the sharing of experiences, their opinions on alternative treatments and their coping strategies. Patients had more opportunity to converse as they exercised in the circuit groups:

*“It’s more communication. I like being with people, and even after doing our exercise and even talking at the same time, you get to know about people and get to know about their illness but we’re still exercising so is quite good.”* (Harry)

The Pilates classes, as ‘taught’ sessions, did not allow the same opportunity, but patients described sharing information afterwards, which helped individuals appreciate personal improvement and enabled sharing of ideas on ways to help themselves. A number of patients also found it helpful and encouraging being with others in a ‘similar situation’. Being with others they perceived as ‘less well’ than themselves motivated continued attendance and also made them value their current abilities.

*“It’s just a wonderful feeling for me … I’m blessed to be with people who are worse than me, in a wheelchair or something and it motivates me more… I’m so grateful that I’m able to do these things… much better than them, but without judging them either.*“ (Amy)

Therapists leading the groups were described as knowledgeable, supportive, protective, encouraging and welcoming; putting patients at ease. Patients felt able to explore their abilities in a safe environment and welcomed the individual programming and adaptation of exercises; lacking confidence or motivation to exercise at home or in a traditional gym. They were appreciative of guidance and being pushed if the therapist felt they had the capability, but also of being given permission to stop if they were tired or had done enough:

*“…this has been the most positive support I have received since my diagnosis.*” (Barbara)

### Taking part in classes

Participants described physical benefits, both in terms of what they were able to achieve or returning to activities of daily living:

*“Even having a bath was out of the question on my own, … but now I can have that on my own… and I even did a bit of gardening in the summer, … walking on my own to the high street … without having to rely on someone … yeah its’ been an amazing experience really.“* (Amy)

The sense of achievement experienced in group sessions motivated continued attendance, despite tiredness or feeling unwell. Patients felt as though they were continuing the ‘fight’, where even minimal progress was important because a sharp decline in ability could be associated with imminent death:

*“And then I’d make sure that everything’s written down, and … recorded,… and … just gradually increase things … The importance I think … is being able to see if you’re not getting any better… and being able to see if you’re getting worse… it would be depressing… but if things start to gradually decrease then at least I can see them gradually decreasing and it’s not like a sharp, … hopefully anyway, it wouldn’t be a sharp, like decrease that makes me worried that, you know, I could die any minute.”* (Jo)

A number of patients relayed how class participation, and desire to maintain improvements, encouraged them to exercise at home, as part of their self-management:

*”But knowing that it’s going to help you … even if I just do it for sort of 15 minutes, I know that anything is better than nothing at all to, to help … try and keep the strength up in my body for as long as possible.”* (Fiona)

Both physical achievements and supportive relationships contributed to psychosocial benefits in improving confidence, independence, and quality of life:

*”Once I came here and my confidence began with my stick… I could see the change in me, the strength I was developing… The last 6 months or so I’ve been walking… to the high street… I wasn’t able to do that before, but now I can very confidently without worrying that I’m gonna fall down.”* (Amy)

For one participant, Jo, seeing herself progress lifted her mood and gave her hope:

*“Even if it was just a tiny, tiny little bit. At least you can say to yourself ‘well I did that little bit more’, so things might be ok.”* (Jo)

Another participant, Fiona felt group interaction had increased her confidence and decreased her shyness. Also initially hampered by shyness, Emma felt group classes had resulted in her being less isolated and improved her self-esteem. She also described the classes enabling her to get on with life, by doing something for herself and empowering her to resume making her own decisions; again facilitating self-management:

*“I think it helps you to sort of, … just get on with being and helping yourself, as opposed to expecting to go to a class and have somebody … direct you, but all of its, is leading up to you making that decision for yourself to sort of keep healthy.“*(Emma)

Whilst Barbara, the single MND patient, described seeing other patients with serious conditions helped her accept her own condition and value what she could still do rather than focus on what she was missing from her daily life. For her, group classes provided an element of welcome distraction and an opportunity for reflection:

*”Gives me a calm space to think about myself… about facing what the future has in store for me.”* (Barbara)

The social value of being able to get out of the house and having somewhere to go was important for the majority. Additional for Jo was regaining a sense of worth and purpose, that had been lost when she could no longer work. Such activity represented normality:

*“I suppose.... it means that I’m able to … just feel like I’m normal, feel like things are normal for me, whereas … being stuck at home all day or the only thing to do is washing up… it makes me feel, like I don’t want to feel, ‘what’s the point’? … You know, and there is a point, and I know there’s a point, and this, doing exercise makes me feel that point. Seeing people makes me feel that point and, and that’s important to me.”* (Jo)

Although participants did describe the physical impact of the classes, none of the patients that mentioned tiredness and/or being achy post-exercise, perceived this as a barrier. They either reported finding energy once they started or feeling better for having done the class. Harry felt the classes helped him through the side effects of chemotherapy treatment.

Overall, all patients described their experiences positively, using adjectives like ‘amazing’, ‘therapeutic’, ‘wonderful’, and ‘having helped immensely’. No adverse events were mentioned. The classes gave hope for the future. For some, this represented a more positive future, whilst for others, the classes enabled participants to feel more able to face the future, and more confident about the level of care they will receive when they become less well.

## Discussion

Previous qualitative research into palliative patient experience of group exercise has largely focused on cancer patients. This study takes a wider view by eliciting the experiences of people with cancer, respiratory diseases and MND, purposefully selected to better reflect a typical modern hospice population. Three main themes were identified ‘perceptions of palliative care and hospice’, ‘supportive relationships’ and ‘taking part in classes’, with patients’ experience primarily centered on the benefits of taking part. Physical gains reported included improvement and maintenance of function, increased strength and improved sleep, in line with existing research into the effect of exercise in advanced disease [2, 24]. The supposition, that every indication that the body still works physically, as well as emotionally and spiritually, is proof of life [25], explains the desire of Jo to make progress each week as she associated decline with impending death. Much of the previous rehabilitation research has focused on functional improvement, using physical outcome measures even when studying progressive neurological conditions where physical function tends to decline. Here we show that maintenance of function can also positively impact upon reported quality of life, complementing previous findings that psychosocial improvements can provide overall benefit even when physical goals are not being achieved [7, 26].

Enjoyment of a sense of comradeship mentioned by one patient, and ‘friendship’ and ‘kinship’ mentioned by another, endorses findings concerning relationships highlighted in a previous study [24]. Although not raised here, the data from Stevinson et al. suggest that small group sizes (e.g. 10 or fewer participants) encourage the building of these relationships over the course of the programme, preventing isolation [24]. Support from others, regarded as a benefit of participating in group exercise, and ‘togetherness’ have previously been recognized as important elements for coping with terminal illness [25]. Sharing personal information, reciprocal concern for each other and the comparing of self to others considered to be in a similar situation enabled a shared identity, established rapport and enhanced relationships [24]. Studies in groups of patients with cancer diagnoses suggest the collective membership enables social support and solidarity among the group [6, 24]. This study extends these findings and indicates this support is also present and important within groups with different cancer and non-cancer conditions. Another aspect not covered in previous literature but revealed through the present study was that the opportunity to share often relied on class format. Circuit classes facilitated by a therapist allowed patients the opportunity to talk as they exercised, whereas Pilates-based classes that were instructed by a therapist did not.

Comparing themselves to others perceived as being ‘less well’ produced feelings of gratefulness within participants, and provided motivation for exercising on the premise that if less well patients were ‘getting on with it’ then so should they. This could be considered an example of the ‘meaning-making process’ used as a coping strategy, where a positive result is found in a distressing situation, such as facing one’s own mortality [25]. Social comparison can have both positive and negative consequences, for example in a study of people with MND, seeing others coping well with the condition was found to provide hope, while at times people were saddened by seeing others with the condition in a worse state [27]. Inspiration to work harder through exercising with others has been previously recognized [24], whereas concern for other patients and how that might affect individuals, highlighted in our findings, is rarely reported. Patient sadness but resignation to the inevitable deterioration of other group members; epitomized in Fran’s statement that “one of these days it’s gonna be us”, is recognized as a factor associated with, and seen as characterizing, death [25]. Interestingly, none of the male participants spoke of sadness or attached any emotion to the prospect of deterioration of group members; supporting the suggestion that men and women may have different needs within groups [28]; men preferring sharing of information whilst women may find intimacy, mutual support and emotional disclosure more important.

Patient appreciation that staff members adapted and tailored exercises, along with the lack of pressure to work harder than they feel able to, expressed in the interviews, corroborates previous data [24]. Some patients desired to be ‘pushed’ or motivated by the therapist in order to test ability and capacity. Rather than valuing this motivation when tired or low in mood [29], patients interviewed were appreciative of encouragement and permission to do less if they were tired. Confidence and trust, considered important aspects to the patient-therapist relationship [30], were evident in comments regarding therapist knowledge, and their handling of situations with inclusiveness, empathy, patience and understanding.

Psychosocial benefits extending to self-empowerment, a desired outcome of palliative rehabilitation was reflected in Emma’s comment on how attending classes helped her take control in making her own decisions about her health, rather than being directed by others. This fits the ‘self-care’ model promoted by the Department of Health [31] that includes self-care of long term conditions. The interviews also confirmed findings of a previous study [24], that physical achievements and supportive relationships contribute to psychosocial benefits such as improved mood, increased confidence and a sense of achievement. Other research has identified that feelings of restricted living and separation, not just from people but also from roles, can result in feelings of isolation, adversely affecting quality of life [25]. This may be why needing to be somewhere and having something to do, when no longer working, gave Jo a desired sense of purpose. It is suggested that such a sense of purpose and participation in activity can redress feelings of isolation and despondency [25].

All these elements fed into psychosocial enhancement, the development of hope and improved quality life [26], as did accomplishing tasks, e.g. exercises, and the support of staff. Patients’ initial focus in agreeing to attend classes was to gain some physical benefit, as described in the lower tier of Maslow’s hierarchy of need [32]. In many cases this led to psychosocial benefit at the higher tiers of the pyramid that patients were not necessarily aware, of or aiming for, at the outset. It is interesting to note that an exercise class provided a supportive environment, with opportunity for personal reflection for at least one patient, and allowed others to feel confident and at ease with accessing other hospice services as they became less well.

Although having treatment, side-effects, fatigue, tiredness and aches experienced from exercise could have presented as barriers to group participation, they were not perceived as such by patients. This may be because the perceived physical and psychosocial benefits outweighed the experience of tiredness and aches, and were perceived as helping with fatigue, maintaining a semblance of normality and helping get through treatment. Exercise is considered to have ‘therapeutic’ benefits [28], as was described by participants in the present study. No adverse events were mentioned. All patients described a positive and enjoyable experience, and a sense of hope, vital to enhancing psychological well-being, was evident in most of the interviews.

There are some limitations to this work. The study explored the experiences of a small number of participants, although such a sample is considered appropriate for a phenomenological approach. A larger sample however may have provided greater ethnic diversity, a more even gender split and a wider variety of diagnoses, as seen in the hospice population. Although the small number of interviews conducted in qualitative research can be said to restrict generalisability of findings, this study has produced core findings that corroborate previous studies. The possibility of ‘sole researcher bias’ in data analysis was addressed through supervision, the testing of codes and themes by two reviewers and discussions with members of the AHP team. Future research may consider the use of focus groups to further explore the benefits of group exercise in advanced illness, and enable added insight into the most important elements of group activities that contribute towards a positive experience.”

## Conclusion

This study explored the experiences of group exercise classes in the hospice setting for people living with cancer and non-cancer diagnoses. All participants expressed a positive experience, and felt they gained physically and/or psychosocially from participating. Acknowledging and sharing these benefits can help shape public perception of hospices and promote such services more effectively], to increase access for all who may benefit. Hospices and other palliative care services should consider introducing similar mixed diagnosis programmes as part of their rehabilitation services.
